# Response to neoadjuvant paclitaxel predicts survival in anaplastic thyroid carcinoma

**DOI:** 10.1002/cam4.5219

**Published:** 2022-09-02

**Authors:** Haruhiko Yamazaki, Kiminori Sugino, Ryohei Katoh, Kenichi Matsuzu, Chie Masaki, Junko Akaishi, Kiyomi Yamada Hames, Chisato Tomoda, Akifumi Suzuki, Keiko Ohkuwa, Wataru Kitagawa, Mitsuji Nagahama, Yasushi Rino, Koichi Ito

**Affiliations:** ^1^ Department of Surgery Ito Hospital Tokyo Japan; ^2^ Department of Pathology Ito Hospital Tokyo Japan; ^3^ Department of Surgery Yokohama City University School of Medicine Yokohama Japan

**Keywords:** anaplastic thyroid carcinoma, neoadjuvant chemotherapy, paclitaxel, prognosis, response

## Abstract

The clinical utilities of paclitaxel in anaplastic thyroid carcinoma (ATC) have been reported. The current study investigated the outcomes in ATC patients treated by paclitaxel as neoadjuvant setting. Furthermore, the prognostic factor for overall survival (OS) and predictive marker for response to paclitaxel were investigated. Records of ATC patients treated by paclitaxel as neoadjuvant setting in our hospital were reviewed. The median OS for the patients with (*n* = 43) and without (*n* = 23) resection were 14.7 (95% CI, 11.0–21.7) and 4.2 (95% CI, 3.0–5.4) months, respectively (*p* < 0.001). Univariate analysis identified the factors of stage (*p* = 0.028), prognostic index (PI) ≥2 (*p* < 0.001), response to paclitaxel (*p* = 0.007), resection (*p* < 0.001), and radiotherapy (*p* < 0.001) to be associated with OS, and multivariate analysis revealed that the factors of PI ≥2 [hazard ratio (HR), 2.406 (95% CI, 1.096–5.281), *p* = 0.029], response to paclitaxel [HR, 0.423 (95% CI, 0.193–0.930), *p* = 0.032], resection [HR, 0.316 (95% CI, 0.129–0.773), *p* = 0.012], and radiotherapy [HR, 0.229 (95% CI, 0.100–0.526), *p* < 0.001] were independent prognostic factors of OS. There were no significant predictive factors for response to paclitaxel in baseline characteristics. PI ≥2, response to paclitaxel, resection, and radiotherapy were independent prognostic factors in ATC patients treated with paclitaxel as neoadjuvant setting. It is important to investigate predictor for response to paclitaxel for improving resectability and prognosis in ATC.

## Graphical abstract

The current study investigated the outcomes in anaplastic thyroid carcinoma (ATC) patients treated by paclitaxel as neoadjuvant setting. Prognostic index ≥2, response to paclitaxel, resection, and radiotherapy were independent prognostic factors in ATC patients treated with paclitaxel as neoadjuvant setting.

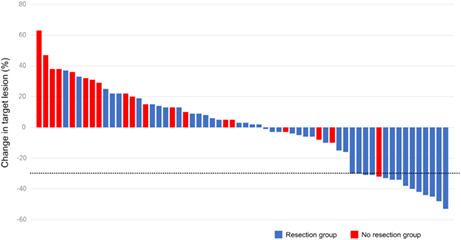

## 1 INTRODUCTION

Anaplastic thyroid carcinoma (ATC) is the most aggressive thyroid carcinoma.^1^ There has been no effective therapy, although a multimodal therapy results in a relatively prolonged survival.^2^ Lenvatinib, a multikinase inhibitor, has recently been used in Japan.^3^, ^4^ The median overall survival (OS) of patients receiving lenvatinib ranges from about 3 to 10 months,^3^, ^4^, ^5^, ^6^ and a more effective drug is desired. The combination of the dabrafenib, which is BRAF inhibitor, and the trametinib, which is MEK inhibitor trametinib became the first targeted regimen approved for ATC based on a previous report.^7^

Paclitaxel is a microtubule‐stabilizing drug with significant activity in various carcinomas.^8^, ^9^, ^10^, ^11^ The clinical systemic activity of paclitaxel in ATC was first studied by Ain et al.^12^ Higashiyama et al. reported that paclitaxel could extend the survival of ATC patients with stage IVB without severe adverse events in Japanese ATC patients.^13^ Based on these previous data,^12^, ^13^ the prospective clinical study to investigate the usefulness of paclitaxel in ATC patients was conducted in Japan.^14^ The study indicated the probability that paclitaxel may be a standardized treatment for ATC patients.

Neoadjuvant chemotherapy has numerous theoretical benefits such as enhancing resectability by chemotherapy‐induced tumor downstaging, preventing delay of receiving systemic therapy, allowing regimen to be continued only in subjects more likely to benefit by assessing response, and identifying patients with rapidly progressive disease during preoperative chemotherapy to avoid nontherapeutic thyroid surgery.^15^ The treatment strategy for unresectable ATC, which has gross extra thyroidal extension and/or distant metastatic lesion, in our institution is that the ATC patients are initially treated by paclitaxel and response is evaluated whether tumor is resectable. The current study investigated the outcomes in ATC treated by paclitaxel as neoadjuvant setting. Furthermore, we investigated prognostic factor for OS and predictive marker for response to paclitaxel.

## 2 MATERIALS AND METHODS

### 2.1 Subjects

**FIGURE 1:**
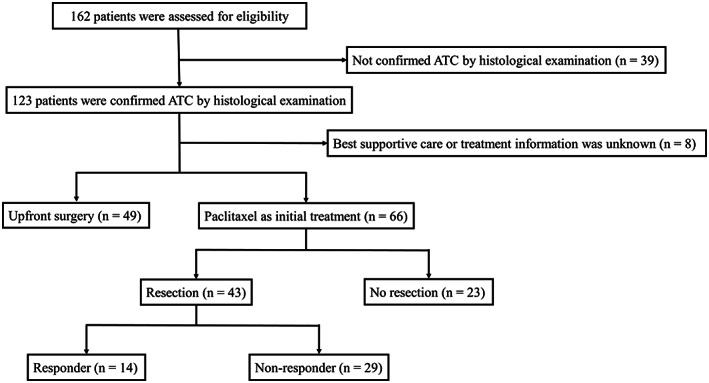
Enrollment and participation flow diagram.

A flow diagram of enrollment and participation is shown in Figure 1. We retrospectively identified 162 patients with ATC between January 2005 and October 2021. A total of 96 patients were excluded from the study (49 underwent upfront surgery, eight chose best supportive care or had no detailed treatment information, and 39 were not confirmed to have ATC by histological examination). Ultimately, we included 66 patients with ATC in the study (Figure 1). Information regarding patient characteristics; laboratory data, including complete blood count (CBC); treatment; and survival were collected. The dose of paclitaxel administered was 80 mg/m^2^ once per week. Response was evaluated after several doses of paclitaxel by computed tomography (CT). The median number of doses before evaluation of resectability was 4 (range, 1–8). The date of evaluation depended on physician's choice because ATCs often progress rapidly. Radiotherapy was performed as adjuvant or controlling cervical residual lesions. Six patients received radiotherapy combined with concurrent reduced dose of paclitaxel (range, 30–60 mg/body). Lenvatinib was administered in recurrent patients who had underwent curative resection or in patients with disease progression after paclitaxel. The median length of follow‐up was 7.7 (range, 0.7–110.2) months for all patients. The study was approved by Ethics Committee of Ito Hospital (approval no. 274).

### 2.2 Definitions

We used the AJCC 8th Edition for ATC staging.^16^ Baseline CBC data were collected when subjects were in stable situation such as before core needle biopsy, or induction of paclitaxel for ATC. The prognostic index (PI) comprises four factors: maximum tumor diameter, acute symptoms, white blood cell (WBC) count, and distant metastasis.^17^ The response to paclitaxel was evaluated by CT using revised Response Evaluation Criteria in Solid Tumors, version 1.1.^18^ The clinical response was classified into four categories: complete response, partial response, stable disease, and progressive disease. The objective response rate was defined as the proportion of patients with complete response or partial response. Inflammatory biomarkers which were associated with survival or anti‐cancer‐drug effect in ATC patients were calculated like previous study.^19^, ^20^, ^21^ OS was defined as the interval from the date of diagnosis to the date of death.

### 2.3 Statistical analysis

All statistical analyses were conducted using EZR (Saitama Medical Center, Jichi Medical University), a graphical user interface for R (The R Foundation for Statistical Computing).^22^ Fisher's exact test was used for comparing categorical variables, and the Mann–Whitney *U* test was used for continuous variables. Kaplan–Meier method was used for constructing OS curves. The factors associated with OS were determined by the cox proportional hazards model. We considered a *p* value of <0.05 as statistically significant.

## 3 RESULTS

### 3.1 Baseline characteristics

**FIGURE 2:**
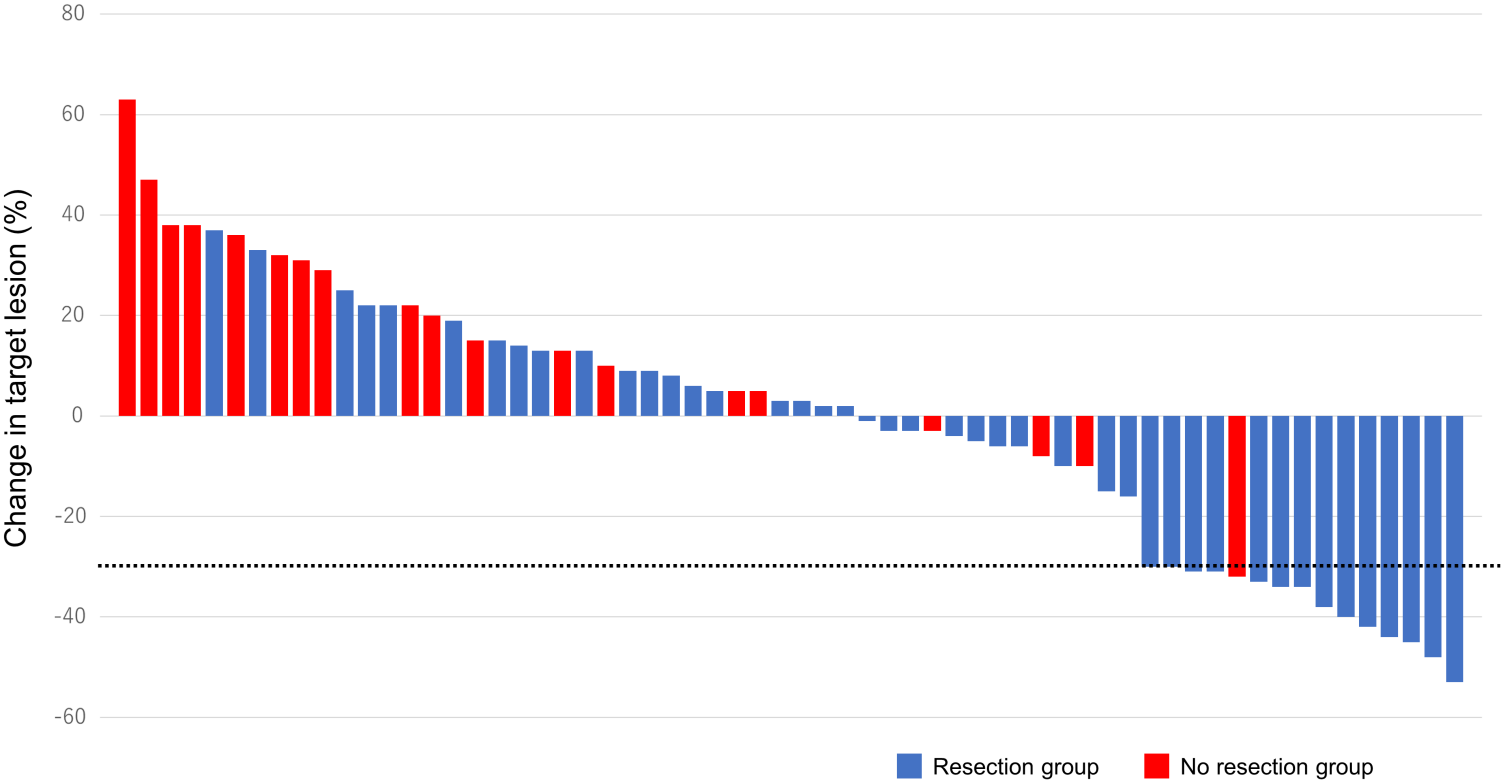
Clinical response to paclitaxel. Change from bas eline in target lesion diameter was determined according to Response Evaluation Criteria in Solid Tumors (RECIST), version 1.1. The black dotted line indicates a 30%decrease, which is the minimumchange needed to qualify for partial response according to RECIST. In all 66 patients, four patients died before assessment of clinical response because their general condition worsened.

Baseline characteristics are shown in Table 1. The study included 24 men and 42 women, with a median age was 69 (range, 39–84) years, a median tumor size was 55 (range, 19–120) mm, a median WBC count was 8185 (range, 4600–65,900) /μl. In patients with stage IVA (*n* = 6), paclitaxel was administered to prevent disease progression until surgery. Two patients had cervical lymph node recurrence with anaplastic transformation. Among the included patients, 43 (65%) underwent resection after paclitaxel (resection group) and 23 (35%) could not (no resection group). Clinical response to paclitaxel was shown in Table 2 and Figure 2. In all 66 patients, the median number of doses of paclitaxel was 12 (range, 2–24). The objective response rate (complete response + partial response) in all 66 patients was 23% (*n* = 15). In resection group, the median numbers of doses of paclitaxel before and after surgery were 6 (range, 1–10) and 8 (range, 0–20), respectively. On the other hand, the median number of doses of paclitaxel was 6 (range, 2–24) in no resection group. Thirty‐eight of 43 patients had no macroscopic residual tumor (R0). In no resection group, 10 patients developed evidence of disease progression. The duration of paclitaxel treatment in these 10 patients was ranged from 1 to 5 weeks. Partial response and stable disease for response to paclitaxel were observed in one and 8 patients, respectively. However, these patients had too large a tumor burden to undergo resection. The remaining four patients died before assessment of clinical response because their general condition worsened. The objective response rate was significantly higher in resection (*p* = 0.012).

**TABLE 1:** Patients' baseline characteristics

	All (*n* = 66)	Resection (*n* = 43)	No resection (*n* = 23)	*p* value
Sex				0.593
Male	24 (36%)	17 (39%)	7 (30%)	
Female	42 (64%)	26 (61%)	16 (70%)	
Age (years), median (range)	69 (39–84)	69 (39–83)	69 (44–84)	0.609
Tumor size (mm), median (range)	55 (19–120)	50 (19–120)	61 (25–103)	0.146
WBC count (/μl), median (range)	8185 (4600–65,900)	8170 (4600–25,200)	8300 (5200–65,900)	0.893
Performance status				0.172
0 or 1	60 (91)	41 (95%)	19 (83)	
≥2	6 (9)	2 (5%)	4 (17%)	
Stage				0.188
IVA	6 (9%)	6 (14%)	0	
IVB	29 (36%)	18 (30%)	11 (48%)	
IVC	29 (52%)	17 (51%)	12 (52%)	
Anaplastic transformation	2 (3%)	2 (5%)	0	
Prognostic index				0.034
0 or 1	23 (33%)	19 (42%)	4 (17%)	
≥2	43 (67%)	24 (58%)	19 (83%)	
Radiotherapy	41 (62%)	33 (77%)	8 (35%)	0.001
Lenvatinib	17 (26%)	11 (26%)	6 (26%)	1

Abbreviation: WBC, white blood cell.

**TABLE 2:** Efficacy of paclitaxel treatment

	All (*n* = 66)	Resection (*n* = 43)	No resection (*n* = 23)	*p* value
Clinical response				
Complete response	0	0	0	
Partial response	15 (23%)	14 (33%)	1 (4%)	
Stable disease	32 (48%)	24 (56%)	8 (35%)	
Progressive disease	15 (23%)	5 (12%)	10 (43%)	
Not evaluable	4 (6%)	0	4 (17%)	
Objective response rate	15 (23%)	14 (33%)	1 (4%)	0.012

### 3.2 Analysis of overall survival

**FIGURE 3:**
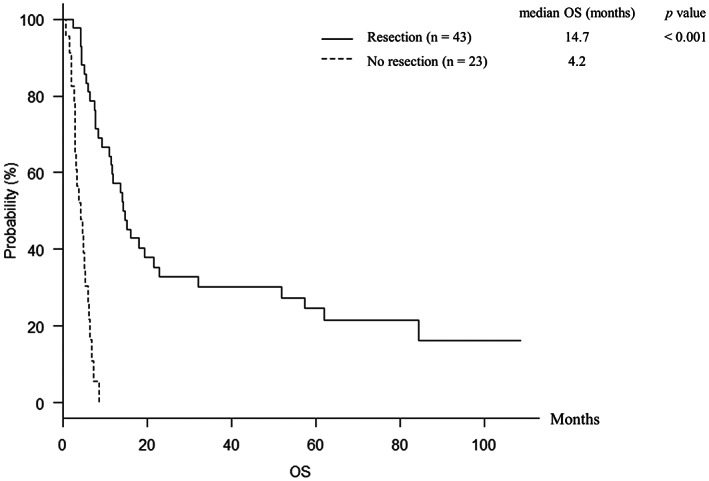
Overall survival for the patients in resection and no resection group after paclitaxel. The median overall survival for the patients in resection and no resection group were 14.7 [95% confidence (CI), 11.0–21.7] and 4.2 (95% CI, 3.0–5.4) months, respectively (*p* < 0.001).

The median OS for the 66 patients was 7.7 [95% confidence interval (CI), 5.9–12.0] months, and the 1‐year OS rate was 37.9%. The median OS for the patients in resection and no resection group were 14.7 (95% CI, 11.0–21.7) and 4.2 (95% CI, 3.0–5.4) months, respectively (*p* < 0.001) (Figure 3). The 1‐year OS rates in resection and no resection group were 57.2% and 0%, respectively. Furthermore, the median OS for the patients with and without response to paclitaxel were 51.9 (95% CI, 7.7–not applicable) and 6.4 (95% CI, 5.0–11.6) months, respectively (*p* = 0.007). Table 3 presents the results of univariate and multivariate analysis for OS. Univariate analysis identified the factors of stage (*p* = 0.028), PI ≥2 (*p* < 0.001), response to paclitaxel (*p* = 0.007), resection (*p* < 0.001), and radiotherapy (*p* < 0.001) to be significantly associated with OS (Table 3). Further analysis was performed by submitting those factors for multivariate analysis. Multivariate analysis showed that the factors of PI ≥2 [hazard ratio (HR), 2.406 (95% CI, 1.096–5.281), *p* = 0.029], response to paclitaxel [HR, 0.423 (95% CI, 0.193–0.930), *p* = 0.032], resection [HR, 0.316 (95% CI, 0.129–0.773), *p* = 0.012], and radiotherapy [HR, 0.229 (95% CI, 0.100–0.526), *p* < 0.001] were independent prognostic factors of OS (Table 3). During follow‐up period, 33 and 22 patients died in resection and no resection group, respectively. Airway obstruction by local tumor caused death in 3 of 33 patients in resection group and 13 of 22 patients in no resection group, and the frequency of airway obstruction was significantly less in resection group (*p* < 0.001).

**TABLE 3:** Analysis of factors associated with overall survival

		Univariate analysis	Multivariate analysis		
Variables	*n* = 66	*p* value	HR (95% CI)	*p* value	1‐year OS (%)
Sex		0.548			
Male	24 (36%)				50.0
Female	42 (65%)				30.6
Age (years)		0.76			
<55	5 (8%)				40.0
≥55	61 (92%)				37.7
Stage		0.028	0.913 (0.468–1.782)	0.790	
Other stages	37 (48%)				47.9
IVC	29 (52%)				25.1
Prognostic index		< 0.001	2.406 (1.096–5.281)	0.029	
0 or 1	23 (33%)				64.7
≥2	43 (67%)				23.9
Response to paclitaxel		0.007	0.423 (0.193–0.930)	0.032	
Yes	15 (23%)				57.4
No	51 (77%)				32.1
Resection		< 0.001	0.316 (0.129–0.773)	0.012	
Yes	43 (65%)				57.2
No	23 (35%)				0
Radiotherapy		< 0.001	0.229 (0.100–0.526)	< 0.001	
Yes	41 (62%)				56.1
No	25 (38%)				5.1
Lenvatinib		0.672			
Yes	17 (26%)				29.4
No	49 (74%)				41.1

Abbreviations: HR, hazard ratio; OS, overall survival.

### 3.3 Analysis of predictive factor for response to paclitaxel

As patients with response to paclitaxel had better prognosis, and responders were more likely to undergo resection, the analysis of predictive factor for response to paclitaxel was performed. The baseline characteristics sex, age, tumor size, WBC count, lymphocyte count, NLR, PLR, LMR, stage, and PI were analyzed. However, there was no significant predictive factor (Table 4).

**TABLE 4:** Analysis of predictive factor for response to paclitaxel

		Responder (*n* = 15)	Non‐responder (*n* = 51)	*p* value
Sex				0.543
Male		4 (27%)	20 (39%)	
Female		11 (73%)	31 (61%)	
Age (years), median (range)		71 (59–82)	69 (39–84)	0.28
Tumor size (mm), median (range)		50 (30–102)	58 (19–120)	0.239
WBC count (/μl), median (range)		8260 (4600–23,300)	7900 (4800–65,900)	0.836
Lymphocyte count (/μl), median (range)		1411 (782–3262)	1613 (528–6486)	0.375
NLR				0.339
Mean ± SD		5.0 ± 1.9	5.0 ± 3.1	
Median (range)		4.7 (1.8–9.0)	3.8 (0.9–14.0)	
PLR				0.327
Mean ± SD		212.9 ± 65.1	196.8 ± 95.4	
Median (range)		211.9 (119.3–355.5)	181.7 (58.6–528.4)	
LMR				0.342
Mean ± SD		2.8 ± 1.2	3.5 ± 1.9	
Median (range)		2.7 (1.1–6.3)	2.7 (1.0–12.0)	
Stage				1
Other stages		8 (53%)	25 (49%)	
IVC		7 (47%)	26 (51%)	
Prognostic index				0.547
0 or 1		6 (40%)	16 (31%)	
≥2		9 (60%)	35 (69%)	

Abbreviations: LMR, lymphocyte‐to‐monocyte ratio; NLR, neutrophil‐to‐lymphocyte ratio; PLR, platelet‐to‐lymphocyte ratio; WBC, white blood cell.

## 4 DISCUSSION

The current study evaluated the outcomes in ATC treated by paclitaxel as neoadjuvant setting. The median OS was 14.7 months in patients who underwent resection after paclitaxel, and it was significantly better prognosis compared with patients who did not.

The clinical trial of weekly paclitaxel for ATC patients by Higashiyama et al. reported that the response rate of paclitaxel was 31%. Furthermore, four stage IVB patients underwent curative resection after paclitaxel.^13^ In Japanese phase 2 study that included 50 ATCs, eight patients who underwent complete resection after paclitaxel survived a median of 7.6 months, significantly longer than those who could not undergo resection (median, 5.4 months).^14^ The response rate to paclitaxel in this study was 23%, and it was similar to the results of the previous study.^14^

Patients who underwent resection after paclitaxel had a significantly better prognosis despite half of these patients having distant metastatic disease, indicating that local control of ATC may improve survival. Kitamura et al. reported that the most common cause of death was respiratory insufficiency (43%), followed by circulatory failure (15%), hemorrhage from tumor (15%), and airway obstruction (13%) in thyroid cancer.^23^ Aggressive local resection can prevent hemorrhage and airway obstruction from the tumor. In fact, the incidence of airway obstruction was significantly less frequent among patients who underwent resection after paclitaxel. In Japanese guidelines, multidisciplinary treatment including radiotherapy, chemotherapy, and molecular‐targeted therapy are considered for stage IVB ATC with a PI of ≥2 and for those with stage IVC.^24^ Palliative surgery is also controversial in the guidelines.^24^ However, since some ATC patients have the component of differentiated thyroid carcinoma, a distant lesion may be from a differentiated carcinoma component. In our institution, primary tumor resection was aggressively performed when a distant lesion was stable during chemotherapy, which maybe led to a better prognosis in some ATC patients. Of course, it is important to consider a patient's general condition when determining whether surgery is appropriate.

Fan et al. revealed that radiotherapy and trimodal therapy (surgery, radiotherapy, and systemic therapy) improved OS.^25^ This study also identified that response to paclitaxel, resection, and radiotherapy were independent prognostic factors. To improve resectability, we should predict patients who respond to paclitaxel. However, there was no association between response to paclitaxel and baseline characteristics including inflammatory biomarkers. Therefore, it would have been difficult to select on the basis of those parameters. Class III beta‐tubulin (TUBB3), which is one of beta‐tubulin isoforms has been reported to be associated with resistance to Taxol‐based chemotherapy.^26^, ^27^ Therefore, there is the possibility that we could predict response to paclitaxel by identifying such molecular marker in tumor tissues. Na et al. showed that ATCs had higher expression of TUBB3.^28^ However, the correlation between the response to paclitaxel and TUBB3 level has not been examined in ATC, and we need further study to identify the predictor for response to paclitaxel.

Response‐guided therapy is considered to be useful treatment strategy.^29^, ^30^ Maniakas et al. reported that the patient management changes could increase survival for ATCs.^31^ In their treatment algorithm, Stage IVB and IVC ATCs receive neoadjuvant BRAF and MEK inhibitors with or without immunotherapy, and surgery is performed if the tumor is considered to be resectable.^31^ As other malignancy, response‐guided therapy probably increases survival of ATCs. In our institution, the unresectable ATC patients are initially treated by paclitaxel, and then we evaluate whether tumor is resectable after several doses of paclitaxel. This strategy seems reasonable based on report from other facility.^31^ In this study, the factor of response to paclitaxel was identified as prognostic factor, and responders more likely underwent resection. However, some patients in resection group died relatively early as recurrent or distant metastatic disease became uncontrollable. We need further study whether we should change the agent from paclitaxel to others, such as lenvatinib, after resection in non‐responders.

Lenvatinib has significant antitumor activity against ATC and may improve the prognosis for ATC compared with weekly paclitaxel.^32^ Cases of surgery after lenvatinib treatment used as neoadjuvant therapy have been reported,^33^, ^34^, ^35^ and lenvatinib is considered to be a good candidate due to its antitumor efficacy. However, the safety of lenvatinib as neoadjuvant therapy has not been established. A Korean single‐center study revealed that adding lenvatinib to paclitaxel and radiation therapy in ATC patients who had progressed on prior therapy could improve outcome and bring tumor volume reduction. The range of tumor volume reduction rates was 2%–69% in 16 ATC patients.^36^ Further investigation is needed whether lenvatinib is useful for neoadjuvant therapy.

At least half of all ATCs have a BRAF V600E or RAS mutation, and the mutation rate of overall BRAF V600E is approximately 30%.^37^ In the latest American Thyroid Association Guidelines, assessment of BRAFV600E mutation should be examined immediately by immunohistochemistry or molecular testing.^38^ Although the cases of ATC patients who have undergone curative resection after neoadjuvant of BRAF and MEK inhibitors have been reported, these agents have not been approved in Japan.^39^ Furthermore, assessment of BRAFV600E mutation has not been performed routinely. Considering such a social background, paclitaxel may be a good agent for neoadjuvant chemotherapy in ATC that have no targetable gene mutation such as BRAF, RET, or TRK.^38^

This study has some limitations. First, this was a retrospective single‐center study. Second, the study had some selection bias in that ATC patients who were not confirmed by histological examination were excluded. Third, there was the possibility that patients who respond to paclitaxel simply had biologically favorable tumors than non‐responders. Although only clinical factors were examined in this study, further study is needed to analyze pathological and genetic factors. Finally, the protocol for the treatment strategy was not established. The number of doses of paclitaxel before surgery or assessment of resectability depended on length of time to surgery or physician's choice. To determine the efficacy of paclitaxel as neoadjuvant chemotherapy, patients' cycles of chemotherapy should be determined prospectively. In this study, the median number of doses of paclitaxel before evaluation of resectability was 4, and this number may be reasonable considering the aggressiveness of ATC. Despite these limitations, however, we believe that the results of this study are important, given that this is the first large study to investigate the prognostic factor in ATC patients treated with paclitaxel as neoadjuvant setting.

In conclusion, PI ≥2, response to paclitaxel, resection, and radiotherapy were independent prognostic factors in ATC patients treated with paclitaxel as neoadjuvant setting. Additionally, patients with resection after paclitaxel had significantly better prognosis compared with patients without resection. It is important to investigate predictor for response to paclitaxel for improving resectability and prognosis in ATC.

## AUTHOR CONTRIBUTIONS

Haruhiko Yamazaki: Conceptualization, data analysis, writing of the manuscript, data curation, and approval of the final manuscript. Ryohei Katoh: Pathological examination. Kiminori Sugino: Conceptualization and data curation. Kenichi Matsuzu: Data curation. Chie Masaki: Data curation. Junko Akaishi: Data curation. Kiyomi Yamada Hames: Data curation. Chisato Tomoda: Data curation. Akifumi Suzuki: Data curation. Keiko Ohkuwa: Data curation. Wataru Kitagawa: Data curation. Mitsuji Nagahama: Data curation. Yasushi Rino: Supervision. Koichi Ito: Supervision.

## CONFLICT OF INTEREST

The authors made no disclosures.

## Data Availability

Data is available upon reasonable request.
